# A New Concept of Work Engagement Theory in Cognitive Engagement, Emotional Engagement, and Physical Engagement

**DOI:** 10.3389/fpsyg.2021.663440

**Published:** 2022-02-14

**Authors:** Stanley Y. B. Huang, Chien-Hsiang Huang, Tai-Wei Chang

**Affiliations:** ^1^Master Program of Financial Technology, School of Financial Technology, Ming Chuan University, Taipei City, Taiwan; ^2^General Education Center, Chihlee University of Technology, New Taipei City, Taiwan; ^3^Graduate School of Resources Management and Decision Science, National Defense University, Taipei City, Taiwan

**Keywords:** authentic leadership, latent growth model, organizational citizenship behavior, task performance, work engagement

## Abstract

The concept of work engagement (WE) has aroused the interest of many scholars. However, there has been limited academic research in examining how authentic leadership (AL) can influence WE, which consequently influences organizational citizenship behavior (OCB) and task performance (TP). In particular, this study divides WE into cognitive engagement, emotional engagement, and physical engagement to fully reflect the engagement theory. This study introduces three dimensions of WE and tests the theoretical model to validate cognitive engagement, emotional engagement, and physical engagement. Empirical testing using a survey of 151 employees of retail travel agencies in Taiwan revealed that the AL can influence cognitive engagement, emotional engagement, and physical engagement, and also OCB and TP. These analysis results can assist vendors to implement OCB and TP through WE and AL.

## Introduction

To increase competitive advantages, contemporary travel agencies should make strategies to inspire the willingness of an employee to put personal resources into work (Ariza-Montes et al., 2018; Basinska and Dåderman, 2019; Langseth-Eide, 2019). Therefore, many studies have a great deal of interest in work engagement (WE) because WE can drive performance and organizational outcomes (Macey and Schneider, 2008; Lee and Huang, 2019; De Carlo et al., 2020). However, the past scholars have put in a lot of effort in identifying the content of WE because WE is ambiguous to be regarded as another positive variable, such as job involvement, emotional commitment, or job identity (e.g., Saks, 2006; Viljevac et al., 2012). In particular, the past study has also developed antecedents and outcomes of WE based on past practitioner literature or past studies rather than the engagement theory developed by Kahn (1990) (e.g., Saks, 2006) because the theory developed by Kahn (1990) was criticized as being too conceptual to measure concretely (Singh et al., 2016). Besides, past studies (Singh et al., 2016; Oh et al., 2018) have proposed that the link between authentic leadership (AL) and WE has been ignored, so this study examines whether AL can affect WE. This study also includes organizational citizenship behavior (OCB) and task performance (TP) as outcomes of WE to confirm its criterion of validity (Lincoln, 2001).

Overall, this study investigates how AL affects WE and how WE affects OCB and TP. This study adopts 151 salespeople of Taiwanese retail travel agencies to address these gaps in the literature, and the research question is as follows:

Can AL affect OCB and TP through cognitive engagement, emotional engagement, and physical engagement of WE?

## Theory and Hypotheses

### Work Engagement Theory of Kahn

Work engagement denotes the degree to which a person shows self-preference in job tasks to promote connections between self and job, which can increase role performance through cognitive, emotional, and physical self-investment (Kahn, 1990). Based on the WE theory, this study believes that the WE theory should be divided into cognitive engagement, emotional engagement, and physical engagement. For example, a person who invests cognitive resources in work (e.g., I ought to work hard) to increase the role performance is not necessarily to put emotional resources into a job (e.g., I am enthusiastic for work) or physical resources into a job (e.g., I actually work hard) at the same time. This study defines cognitive engagement as a level of focus, concentration, engrossment, and the focused intensity for a job, and it includes research performed by Rothbard (2001) for absorption as its representative variable. This study defines emotional engagement as joy for a job, and it refers to a past study (Russell and Barrett, 1999) to be its representative variable (i.e., core affect). This study defines physical engagement as work intensity (density of energy consumed by work) that has been developed by the past study (Brown and Leigh, 1996). The absorption, core affect, and work intensity have also been adopted to measure WE in the past study (e.g., Huang et al., 2021). In addition, according to the WE theory (Kahn, 1990), he found that safety, meaningfulness, and availability are the three important driving factors of WE.

### Authentic Leadership and Work Engagement

Authentic leadership denotes that a leader adopts balanced handling, internalized ethical views, relationship transparency, and self-awareness to guide subordinates toward loyalty, trust, and performance (Walumbwa et al., 2008), and it can affect WE through safety, meaningfulness, and availability, which are the three antecedents proposed by Kahn (1990). First, a leader can change the self-concept of a subordinate to meet organizational values, so the subordinate who perceives a high-level AL should perceive high-level meaningfulness (Huang et al., 2021). In other words, the subordinates believe that their self-concept is consistent with their organization by the AL and it will yield a high level of meaningfulness for the job (Kahn, 1990). Second, internalized ethical views and relationship transparency can strive the subordinates to perceive trust and openness, so the subordinates should feel a high level of safety in their organization. Finally, previous studies have proposed that AL can increase the self-development and self-confidence of the employees (Walumbwa et al., 2008), and the subordinates are self-confident, positive, and well-adjusted to believe in efficacy within an organization to achieve high levels of availability because of AL. In fact, past studies have examined similar connections between AL and absorption, core affect, and work intensity (e.g., Smithikrai and Suwannadet, 2018; Duarte et al., 2021; Huang et al., 2021).

*Hypothesis 1:* AL can influence absorption.

*Hypothesis 2:* AL can influence core affect.

*Hypothesis 3:* AL can influence work intensity.

### Work Engagement, Organizational Citizenship Behavior, and Task Performance

The past study has argued that engaged employees have a high-level emotional attachment to their organization (Sonnentag, 2003), and engaged employees should exhibit high levels of OCB (Organ, 1988). Besides, the engaged employee puts multiple resources (i.e., emotional, physical, and cognitive resources) on work and should exhibit a high level of TP. Kahn (1990) also argued that there may be a connection between the WE and performance. In fact, past studies have examined similar connections between WE, OCB, and TP (e.g., Khusanova et al., 2021; Martinez et al., 2021).

*Hypothesis 4:* Absorption can influence OCB.

*Hypothesis 5:* Core affect can influence OCB.

*Hypothesis 6:* Work intensity can influence OCB.

*Hypothesis 7:* Absorption can influence TP.

*Hypothesis 8:* Core affect can influence TP.

*Hypothesis 9:* Work intensity can influence TP.

## Methodology

The framework of this study is from AL to OCB and the TP through the mediating role of cognitive engagement (absorption), emotional engagement (core affect), and physical engagement (work intensity).

### Sampling

This study adopts snowball sampling to collect data, and 160 questionnaires were distributed to the employees of a retail travel agency in Taiwan. We finally collected the data of 151 employees of a retail travel agency in Taiwan to assess AL, absorption, core affect, work intensity, OCB, and TP. In addition, to alleviate memory bias (Morewedge et al., 2005), the respondents were not notified that they may be requested to fill in the absorption, core affects, work intensity, OCB, and TP scale to reduce the effect of predicting the future.

### Measures

All self-report questionnaires used a 7-point Likert-type scale and a backward translation technique (Reynolds et al., 1993).

Authentic leadership was assessed using the scale developed by Walumbwa et al. (2008). WE was assessed using the core affect proposed by Russell and Barrett (1999), work intensity proposed by Brown and Leigh (1996), and absorption proposed by Rothbard (2001). OCB was assessed using the scale developed by Lee and Allen (2002). TP was assessed using the scale developed by Williams and Anderson (1991).

### Data Verification

This study adopts the analysis of confirmatory factor to test the five constructs with its items, including the AL, absorption, core affect, work intensity, OCB, and TP, and the analysis results meet the suggestions of Fornell and Lacker (1981).

#### Analysis Results

The analysis result is in Table 1. First, the AL would significantly result in absorption (*B* = 0.27, *p* < 0.01), core affect (*B* = 0.35, *p* < 0.01), and work intensity (*B* = 0.31, *p* < 0.01), which supported hypotheses 1–3. Second, higher levels of absorption (*B* = 0.27, *p* < 0.01), core affect (*B* = 0.29, *p* < 0.01), and work intensity (*B* = 0.33, *p* < 0.01) would significantly influence the higher levels of OCB, which supported hypotheses 4–6. Third, higher levels of absorption (*B* = 0.25, *p* < 0.01), core affect (*B* = 0.21, *p* < 0.01), and work intensity (*B* = 0.36, *p* < 0.01) would significantly influence the higher levels of TP, which supported hypotheses 7–9.

**TABLE 1:** Analysis results.

Hypothesis		Coefficient
Hypothesis 1	AL can influence absorption	0.27**
Hypothesis 2	AL can influence core affect	0.35**
Hypothesis 3	AL can influence work intensity	0.31**
Hypothesis 4	Absorption can influence OCB	0.27**
Hypothesis 5	Core affect can influence OCB	0.29**
Hypothesis 6	Work intensity can influence OCB	0.33**
Hypothesis 7	Absorption can influence TP	0.25**
Hypothesis 8	Core affect can influence TP	0.21**
Hypothesis 9	Work intensity can influence TP	0.36**

*p < 0.05; **p < 0.01.

#### Alternative Models

**FIGURE 1:**
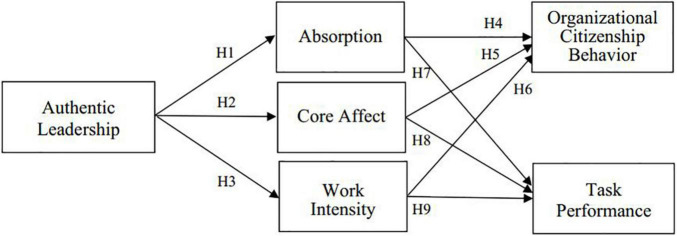
Research model of this study.

To validate the theoretical model of this study (see Figure 1), three alternative models (Alternative Models 1–3) were used. According to Alternative Model 1, there is no relationship between the AL and the absorption, core affect, and work intensity development at Time 1. In other words, the alternative model assumes that this study does not significantly attribute increases of a model in the absorption, core affect, and work intensity development to the AL at Time 1. The fit index of model 1 is significantly lower than the theoretical model of this study (Δχ^2^ = 151, *p* < 0.01). However, it is also possible that the AL directly increases not only the absorption, core affect, and work intensity development but also the OCB and TP development. For example, an employee who receives a high-level AL at stage 1 may also engage in the absorption, core affect, work intensity, OCB, and TP development at the same time rather than in a causal capacity. To detect this possibility, this study builds Alternative Model 2 by adding five paths from the AL to the absorption, core affect, work intensity, OCB, and TP. The χ^2^ value of Alternative Model 2 was higher than that of the theoretical model of this study (Δχ^2^ = 129.45, *p* < 0.01). Besides, another possibility for the data is that the absorption, core affect, and work intensity development may not fully mediate the relationship between the AL at Time 1 and the OCB and TP. Two paths were included from the AL at Time 1 to the OCB and TP development in the theoretical model of this study to form Alternative Model 3. The χ^2^ value of Alternative Model 3 was also higher than that of the theoretical model of this study (Δχ^2^ = 121, *p* < 0.01). These analysis results support the proposed model of this study.

## Discussion

This study shows a theoretical framework to explain why AL can influence OCB and TP through WE.

### Theoretical Contribution

This is an exploratory study that examines the relationship between AL and WE through the three domains of WE. In particular, this study demonstrates the other path that the AL also influences the WE when the follower is within an openness and truthfulness environment. Second, previous researchers (Singh et al., 2016) have believed that AL is an important resource for WE, but the empirical evidence is examined in this study. Finally, this study divides WE theory (Kahn, 1990) into cognitive engagement, emotional engagement, and physical engagement and detects three specific variables (i.e., absorption, core affect, and work intensity) to fully reflect the content of these three domains, which can provide sufficient nutrients for WE literature development and advance application in practices.

### Practical Contribution

First, this study provides an effective management mechanism (i.e., AL) to increase organizational performance (i.e., OCB and TP), and the vendors of travel agencies should invest main resources in AL. The leadership connotation of AL must be incorporated into the annual supervisor education and training to increase the AL ability of company supervisors because AL can increase not only the WE of an employee but also the OCB and TP.

Second, this study suggests that supervisors can adopt AL to influence their subordinates to view their roles as a cross-border perspective rather than a job description. For example, these supervisors can inspire their subordinates by giving true feedback about their contribution to organizational performance through the balanced handling, internalized ethical views, and transparency of AL (Walumbwa et al., 2010).

### Limitations and Further Research

First, this study demonstrates a possible path on how AL can continue to explore its effects on different outcomes. Further study should explore broader outcome variables to advance the AL literature. Second, the sample of this study is Taiwan travel agencies, and further study should examine different contexts.

## Conclusion

This is a pioneer study that examines how AL influences OCB and TP through the three dimensions of WE. In fact, WE has been examined in various disciplines, but few studies have explored the nature of WE with cognitive engagement, emotional engagement, and physical engagement. Therefore, this study can not only promote the literature development of WE but also provide a way for companies to implement WE.

## Data Availability Statement

The original contributions presented in the study are included in the article/supplementary material, further inquiries can be directed to the corresponding author.

## Author Contributions

All authors listed have made a substantial, direct, and intellectual contribution to the work, and approved it for publication.

## Conflict of Interest

The authors declare that the research was conducted in the absence of any commercial or financial relationships that could be construed as a potential conflict of interest.

## Publisher’s Note

All claims expressed in this article are solely those of the authors and do not necessarily represent those of their affiliated organizations, or those of the publisher, the editors and the reviewers. Any product that may be evaluated in this article, or claim that may be made by its manufacturer, is not guaranteed or endorsed by the publisher.
